# Supplementary material for: Patterns of Intron Gain and Loss in Fungi
Source: PLoS Biol. 2004 Nov 30;2(12):e422. doi: 10.1371/journal.pbio.0020422 (PMC532390; doi:10.1371/journal.pbio.0020422)
Supplement: Table S1 — Also available at http://genes.mit.edu/NielsenEtAl/. (4.3 MB ZIP). [file pbio.0020422.st001.zip › NielsenEtAl/html/339.html]

AN1002.1.NCU07698.1.MG07150.1.FG09579.1


```
 CLUSTAL W (1.82) Multiple Sequence Alignments - Introns Inserted


Sequence 1: NCU07698.1	1074 aa
Sequence 2: MG07150.1	1054 aa
Sequence 3: FG09579.1	1038 aa
Sequence 4: AN1002.1	916 aa
Alignment Length: 1118 aa
Number Identitical Residues: 350 aa
Alignment Score (without introns) 20557


MG07150.1 	-MADFERTVMEHYQITSLTPEVWPQEKDN-SDASEGEEDSNDKKAKLNRRKSRYQALERA
NCU07698.1	MAADTERTILDFYQVSSLNLVEWPAEKDNNSDVSDDE----TAKQKANRRKSRFQALERV
FG09579.1 	-MADMERTILDFYQIPTLYPTEWPAEKDLENLDEDGED---EKGAALKRRQSRYQALERA
AN1002.1  	----------------------------------------------MKGNNSR-------
          	                                               : .:**       

MG07150.1 	VSTRSSVVPGSEKTANGIGNLVQRDEPDPLGTTDSVVRTLKQYGVPLQDDPRLR~NRFLL
NCU07698.1	VSNRSSVVPGSETSASGVGNLVQRDEPDPLGTTDSVVRTLKQMGVPLQDDSKLR1NRFLL
FG09579.1 	VSQRKSNIPRGDDQG-GVGNIVQKDEPDPLGTTDSVVRTLKHLGLPLQDDHRLR1NRFLL
AN1002.1  	------------------ENLVKVDEPDPLGSTSSVLNALKKRGLPVAEDSRLR~NRFLL
          	                   *:*: *******:*.**:.:**: *:*: :* :** *****

MG07150.1 	SSTTFSPALFLSQMHASADTNQLLMGLDALSQSIDQKSASLKVLVESNFERFVRAKATID
NCU07698.1	SSTTFSPALFLSQVHATDSTEALLNGLEVLNQSIDQKSASLKVLVESNFERFVRAKATID
FG09579.1 	SSTTFSPALFLSQMHATADTRGLLTGLDVLSQSIDQKSASLKVLVETNFERFVKAKATID
AN1002.1  	SSTGFSPALFLSQVHSDASIESLINGLNVLSQSIDQKSASLKVLVEANFERFVRAKATID
          	*** *********:*:  . . *: **:.*.***************:******:******

MG07150.1 	NVYKEMKYRGAEPPAPPT-ARARAHSRHTSRNSFRAGSGNINLTAPLTPQPPDPRKKNAL
NCU07698.1	NVYKEMKYRGAEPAAP----RARAHSRHASRNSFQSTSAAGGLANSLGP-TIDPRKKNAL
FG09579.1 	NVYKEMKYRGAEPPDPNNPGPSNAAQRRSYRNSMVG--AGLGINNHLTSPGTDTRKKNAL
AN1002.1  	SVYTEMRNQGRE--------QEQVAQRRSIG---HVRSISGAKQGPLSSINSSKFRKNAL
          	.**.**: :* *          .. .*::        :        * .   .  :****

MG07150.1 	TKESEYGVTGIKTPLLDVSAKAEDVWGPALGGQEREENLKTVANSLGRFGEYVETSAAIA
NCU07698.1	IRESEYGVMGVKAPLLDVSAKAEDFWGPALGGREKEEHLKTVGSSLDTYKDYVEISAALA
FG09579.1 	TKESEYGVLGIKGPLLEVSAKAEDVWGPALGGREKEENLKTVSNHLTRFKDYVELSTSIA
AN1002.1  	LKESDYGVKGIWAPLTEASVKAEEVWGPALSGREREQMLKSVIDSMERRREVYEIGGHLS
          	 :**:*** *:  ** :.*.***:.*****.*:*:*: **:* . :    :  * .  ::

MG07150.1 	DSIKRKDHESLIEEYSKARRFADEARRIAQQLGST--PPSDSQLYQILLAARMWHDVEEQ
NCU07698.1	ESIKRKDHESLVEEYNRARRFADEAKQIAQNIGSG--EPTEAQLYKILLAARMWHDVDEQ
FG09579.1 	DSIKRKDYESLVDEFTRARRIADESRQLAEELGEE--TPTEPQLYQLLIAGRMWHDVDQQ
AN1002.1  	KSIKQKDYESVFEQYRKARALTQEAKNIADIAGSEGRPLTDEETYVILALGRMWIDVDQQ
          	.***:**:**:.::: :** :::*::.:*:  *. .   :: : * :*  .*** **::*

MG07150.1 	ISAFKRTVWRRLSALHNVSKSDG-PGQPQDQHMELISLLLELGAEDNPIYFWLLSRYDNL
NCU07698.1	IQLFRREVWRRLVSPQAMAKSDATPGQAHDQHMDLITLLLELGVEENPIWAWLQSRVAYL
FG09579.1 	IRMFKRDVWKRLVTLYTVSRTDGPSGRIQDQHMELIGLLLELGVDDNPIWVWLLSRYDHL
AN1002.1  	IQGFKRDLWRRLSEAPSTS-TRITTSGPIEEYMELIGALLELGVDDNPIWVWLLSRYDYL
          	*  *:* :*:**      : :  ...   :::*:**  *****.::***: ** **   *

MG07150.1 	KGKIQSYADRAKVEIEVLRRRLSSSERPAPRAIGSYLRALGRQSIDSRPTNYDSGNIIEL
NCU07698.1	KSRIQSTAEKSKVEIEVLRRRLANVEKPSPQSIASNLRGLGRQSIESKTPTFDSAEVLDV
FG09579.1 	KSKIQSTADHMKVEIEVLRRRLASNDKPNPSTIATHLRALGRPVIEDKPMTFDSPEVVEL
AN1002.1  	RAKIKAFCERGKVEIEILRRRLASGAEPTPQEVASYLRRTPQDSSTGPAHLPDTDQVIEL
          	:.:*:: .:: *****:*****:.  .* *  :.: **   :    . .   *: :::::

MG07150.1 	WEKMLMFLSTLLSPQG-ILAEVVEFWQTVQGFLDGKIQRSLPTGYNGESLVHHQLSEDWA
NCU07698.1	WETIVAFMNNLMSPQG-ILGEVVEFWQTVQGFMDGNTQRTLPLGYKGESQGHHRLTQQSS
FG09579.1 	WEKMLSFLTSLLSSEG-ILGEVVEFWQTVQGFINGSAQAALPVGYRGESRHHHELSQQSI
AN1002.1  	WECVHTYLNRLLSSQGGILGEILDFWEVAQSFIDGNKQKLLPVGFEGESRKHHKFSSSDV
          	** :  ::. *:*.:*.**.*:::**:..*.*::*. *  ** *:.***  **.::..  

MG07150.1 	SNLQKGTVELVEMVREHVFIFFAGPPPEDISLLFSPM-PLSPKTP-MSTSLGGALSPTAY
NCU07698.1	QELQRATVELVDMIREHVLSFFTGPPPEDLSALVSPL-PSTPNTPNPSTTPGSAIQTTTL
FG09579.1 	NELRKGSVELVDLLREHTYSFFVDMPPEDVSLLFSPL-PKTPVPG----TPG-SDSLASP
AN1002.1  	RDLQKGLIELISLVREGVLSLFAEAPVEDVSLLTSPISPSSPSSP---VSLG-----VTP
          	 :*::. :**:.::** .  :*.  * **:* * **::* :* .     : *     .: 

MG07150.1 	RDPRFNLDPNNLPPPSPKRGEAWEKFAFWPPWSNSVSAVHYLSKMLALIGSGASEMAAIA
NCU07698.1	RVP-INLDPNNLPPPSPKRGEAWEKFAFWPPWSNSISGAHYLAKMLTLVGSGASDMASLA
FG09579.1 	RDPRFNFDPNNMPPPSPKRGEVWEKLAFWPPWSNSISGVHYLSKMLTLVGAGAGDLACIE
AN1002.1  	TESRFKLDPKNIPFPTPKRGEPWEDYAFWPPFSNSLSGVNYLGQFLIIIGAAAGEMTTLE
          	  . :::**:*:* *:***** **. *****:***:*..:**.::* ::*:.*.::: : 

MG07150.1 	PVGLGDAQELELLKTL~VNVSRERCVVALCAAWNKDAETIKHVEDWNRAPEDRDVTKMPA
NCU07698.1	PVGKGDPSELEQLRSL~VNATRERCVTALCAAWNRDSENIKYVEDWNRSPTRKDVTKMPA
FG09579.1 	PVESGEGQVLDRLRSL~VGGSRDRCVTALCAAWNRDAENIKFVEDWQRSVEMGDVTRMPA
AN1002.1  	PVSS-SSTSQELLRGL0N------------------------------------------
          	**   .    : *: *                                            

MG07150.1 	CFSAFEGTLLAGMQKILYIPEASAKAGAEDIVTAPPTKLLQMVRSQYVTTLYKALSGMVE
NCU07698.1	SFAAFERALLSGMQKILYVSEAMTKPGAEDIVLPPATKLLQMVRSQYVTTLYKALHGMVE
FG09579.1 	TFAAFEGSVLSGMQKILYIPDAMSRSGSGDIVLPPPTKLLQMVRSQYVTTLYKALSGMVE
AN1002.1  	-------AIVSGLQKILYMSEAMAKPGTVTVVTQPPTKLLQMVRREFISSIEKALGGLVE
          	       ::::*:*****:.:* ::.*:  :*  *.******** :::::: *** *:**

MG07150.1 	NAERSIKKSD-DEWTTDPEVLGATSAANSRVTSLRMSSGTSSISVGSGTINAGDR0NVRM
NCU07698.1	NAELPIKKPD-DDWTTDANDFVLVNNP----SRLRVST------IGGATIDAGDS0NVRM
FG09579.1 	NAERSLKKTE-DDWSIDDESSLASAAAPS--------------MTGKGALDSGDR0NVRM
AN1002.1  	TAEHPTTREENDEWSVSEATAVVRNSNGS------------SASLAADAVDSQNR0NVRI
          	.** . .: :.*:*: .           :            :   .  :::: :  ***:

MG07150.1 	LLTLSNLQRLRSDLVPSLNTQFENAFSVKLTDETKTIRDVLGQIDARLFQSYTRPSIETL
NCU07698.1	LLTLSNLQALRSEVVPNLNTQFENAFSVKLTDETKTIRDVLGQIDARLFQSYTRPSIESL
FG09579.1 	LMTLSNLSSLQSQVVPSLNLQFENAFSVKLTDESKKIREVLGQIHDRLFQSYTKQSIERL
AN1002.1  	LLTLSNIKAFQMDLVPQLIANFEASFSVTLTDEAKLIRQVLDEVEQRLFRSYTEPTITNL
          	*:****:. :: ::**.*  :** :***.****:* **:**.::. ***:***. :*  *

MG07150.1 	RGIIRAGVTAADWAPANGAKPREARPYVYEVLLTLVLVHSQVSTTASS------------
NCU07698.1	RRIIRAGVTASDWPPPSGQKPREVKPYIYEALLDLVLVHTQVSTTAAS------------
FG09579.1 	RDVIQSGISAPDWAPGQGQRTQVAKPYVYDALLTLVLVHSQVSTTAPA------------
AN1002.1  	KTIITEGVTSPTWEPTTS-RPEQVRPYVYNALLALVLVHTEISTTIPSTSSSTSSRSAAS
          	: :*  *:::. * *  . :.. .:**:*:.** *****:::*** .:::::::: ::::

MG07150.1 	-------LTTQVLSYLLEQTSRELLEAFKTRPRYDLSTLLQAALDVEFAAQTLQHYTTQR
NCU07698.1	-------LTSQVLSFLLEQTSRELLEAFKTRARYDLGMLMQATLDVEFVAQTLNHYTTDR
FG09579.1 	-------LTVEVLSFLLEQTSTQLLEAFRRRPRYTLEALMQATLDVEFVAQTLSHYTSER
AN1002.1  	SASGQSPLLTIVLTHLLTQVCTALVNAFNLRASYSLNALIQATLDTEFIAQTMSQYSSEE
          	:::..:.*   **:.** *..  *::**. *. * *  *:**:**.** ***:.:*:::.

MG07150.1 	AKDLQNQIYQEIESRSDRDAVLNMQNELPELRSLLKRLRDASKN~EFACFKKVKRSN---
NCU07698.1	ASELQSAVYQELDSRTDNDARTRLQAELPEMRAVLKRLREASKS~EFACFRKPKKTTGPA
FG09579.1 	ASELQSQIYQELDARTDNDARARLQGELTEMRTVLKRLRDASKN~EFACFKKPKRPG---
AN1002.1  	ASAVQSQIYVELDQRTTHEARARLQSELGEMRGILKRLRERTKG1RLPIETTKQ------
          	*. :*. :* *:: *: .:*  .:* ** *:* :*****: :*. .:.   . :      

MG07150.1 	-------------SAET------ERKDSV-------------
NCU07698.1	ATAGSSAGSAAMLAAQVGDMSGLERTDTRDTGRSYGTTGSER
FG09579.1 	-------------HGPS-------RNNSGISGISA-------
AN1002.1  	------------------------------------------
          	
```
